# The Great War: VII. Sanitation

**Published:** 1918-05-04

**Authors:** 


### VII.—SANITATION.

SANITATION and the prevention of disease compete for first place with such equivocal successes of the war as counter battery work, Lewis guns, aeroplanes, motor lorries, and rations.

The success of sanitation has been greatly aided by the education of those mainly affected by its ordinances—the rank and file of the Army, and more especially the officers.

In the old days of dirt, disease, and death, sanitation was unpopular in the Army, it was unpleasant and troublesome, and consequently unorganised; until quite recently it was considered sufficient to throw in sanitation with the military duties of an orderly man under the supreme supervision of an orderly corporal or sergeant. There are now schools of sanitation, the courses of instruction of which are regularly attended by officers, N.C.O.s, and men, and sanitation has been added to the compulsory subjects for the examination of junior officers for promotion.

In every unit detachments of men are employed exclusively on sanitary duties and others on the care of water, all personally supervised by the officers. During the war also sanitary sections and sanitary squads, which are field units, have materialised and progressively improved.

Sanitation is mainly concerned in the proper disposal of dirt in its widest sense, the dirt of the dictionary, or "matter misplaced."

In a large army in the field there is a wonderful scope for the practice of cleanliness, affecting the soil, the water supply, the food, the person, the clothing, and the quarters in which it lives, whether it be castle, "dug-out," or "bivouac."

ORGANISATION IN THE FIELD.

A General Officer Commanding is responsible for the sanitary condition of the area occupied by his command and for the prevention of disease among those serving in the area, and the commanders of units in areas are similarly responsible to the G.O.C.s of their brigades and divisions.

The Director of Medical Services is the technical adviser of a General Officer Commanding an army, and similarly Deputy-Directors and Assistant Directors are the advisers of the General Officers Commanding corps and divisions.

The officers of the Royal Engineers attached to the Headquarters of General Officers Command ing are also concerned in sanitation, as they control the material and skilled labour required to give effect to the recommendations of the medical officers.

The Field Sanitary Service comprises:—

(*a*) Specialist sanitary officers.
(*b*) Sanitary sections and squads.
(*c*) Regimental sanitary detachments.
(*d*) R.A.M.C. *personnel* attached to units for water duties.

SPECIALIST SANITARY OFFICERS.

Specialist sanitary officers are allotted to General Headquarters, and to the headquarters of armies, corps, and divisions; they act as Staff officers to the responsible Medical Directors.

SANITARY SECTIONS.

A Sanitary Section consists of an officer and twenty-five other ranks, selected, as far as possible, on account of their previous experience of sanitary duties in civil life. Before the war Sanitary Sections were authorised for the lines of communication only, but one of the earliest improvements in the Medical Services attributable to the war was the allotment of one of these units to each division. In this position they rendered

☛ Previous articles appeared on Feb. 9 and 23, March 9 and 23, and April 6 and 20, p. 49.

**THE GREAT WAR**—(*continued*).

invaluable services and became very popular. They, however, suffered from the disadvantage of constant change of areas conformably to the movements of the divisions. After mature deliberation, therefore, a change was decided upon, and in the face of considerable regrets these units were planted permanently in areas to maintain continuity of cleanliness irrespective of the movements of troops, even as a lodging-house is maintained for the coming and going of guests.

A COMPREHENSIVE PROGRAMME.

The duties of a sanitary section include:—

(*a*) The execution of skilled work in connection with the disinfection of infected houses.

(*b*) The registration and marking of usable wells and of water points, the chlorination of drinking water, and the closure, fencing, and placarding of dangerous sources.

(*c*) The maintenance of a nucleus workshop for the manufacture of standard patterns of sanitary appliances.

(*d*) The establishment of nuclei for a continuous system of conservancy and scavenging.

(*e*) The provision of sanitary inspectors.

(*f*) The disposal of horse litter and the prevention of flies.

(*g*) The construction and maintenance of district latrines, urinals, and incinerators.

(*h*) The protection of food from flies, dirt, and dust.

JACKS OF ALL TRADES.

The *personnel* consists of one officer and twenty-five other ranks. The officers were, until recently, medical officers; owing, however, to the demand for their services by the sick and wounded, they have nearly all been replaced by those who have received no medical training; some of them are sanitary engineers or architects.

The men are carefully selected from a reliable class, some have been municipal sanitary inspectors, and others skilled workmen; such previous training and experience are useful; but handy men of fair education soon adapt themselves to requirements, and eventually each one, like the Rajah of Bhong, "can do almost anything."

All sorts and conditions of labour, skilled and other, is, according to circumstances, tacked on to the establishment; in many instances a party of fifty labourers from an Employment Company is permanently attached to the unit, and very useful they are—as a rule doled out in small parties, each under one of the handy men, to assist town or area majors, as the municipal authorities of villages or ruins under military control are called, or to some unit in difficulties with their sanitary arrangements.

During an advance they are invaluable in preparing billets in new territory for the troops who are too fatigued to help themselves.

The ranks of this unit are also continually augmented by fatigue parties from regiments, by skilled pioneers, and by parties of Royal Engineers.

The equipment includes a box of carpenter's tools and disinfecting apparatus of various kinds.

The Sanitary Section is everybody's friend and helper; it is under the administration of the Deputy-Director of Medical Services of the corps in whose area its headquarters happens to be located, but it performs duties in divisional areas for which the G.O.C. of a division is responsible, and so works in conjunction with his Assistant-Director of Medical Services. The activities of the sanitary section are so diverse and so essential that it is difficult to select the most important; it is probably that of the sanitary inspectors. A sanitary area corresponds approximately with that of a division, and it is usually divided into four districts for purposes of inspection; two, limited by the two halves of the front line; one, the reserve area; and the fourth, the back area; to each of which an inspector is allotted. Each inspector is provided with a special pass, and wears a yellow arm-band bearing the letters S.S. in red, and if he does not happen to be a non-commissioned officer he is elevated to that rank without the pay.

(*To be continued.*)

